# Matrix metalloproteinases as therapeutic targets in breast cancer

**DOI:** 10.3389/fonc.2022.1108695

**Published:** 2023-01-19

**Authors:** Mi Jeong Kwon

**Affiliations:** ^1^ Vessel-Organ Interaction Research Center (MRC), College of Pharmacy, Kyungpook National University, Daegu, Republic of Korea; ^2^ BK21 FOUR Community-Based Intelligent Novel Drug Discovery Education Unit, College of Pharmacy and Research Institute of Pharmaceutical Sciences, Kyungpook National University, Daegu, Republic of Korea

**Keywords:** matrix metalloproteinase (MMP), breast cancer, therapeutic target, tumor microenvironment, MMP inhibitor, immune regulation, immunotherapy

## Abstract

Matrix metalloproteinases (MMPs) are the most prominent proteinases involved in tumorigenesis. They were initially recognized to promote tumor progression by remodeling the extracellular matrix through their proteolytic activity. However, accumulating evidence has revealed that some MMPs have protective roles in cancer progression, and the same MMP can exert opposing roles depending on the cell type in which it is expressed or the stage of cancer. Moreover, studies have shown that MMPs are involved in cancer progression through their roles in other biological processes such as cell signaling and immune regulation, independent of their catalytic activity. Despite the prognostic significance of tumoral or stromal expression of MMPs in breast cancer, their roles and molecular mechanisms in breast cancer progression remain unclear. As the failures of early clinical trials with broad-spectrum MMP inhibitors were mainly due to a lack of drug specificity, substantial efforts have been made to develop highly selective MMP inhibitors. Some recently developed MMP inhibitory monoclonal antibodies demonstrated promising anti-tumor effects in preclinical models of breast cancer. Importantly, anti-tumor effects of these antibodies were associated with the modulation of tumor immune microenvironment, suggesting that the use of MMP inhibitors in combination with immunotherapy can improve the efficacy of immunotherapy in HER2-positive or triple-negative breast cancer. In this review, the current understanding of the roles of tumoral or stromal MMPs in breast cancer is summarized, and recent advances in the development of highly selective MMP inhibitors are discussed.

## Introduction

Breast cancer is the most frequent malignancy and the leading cause of cancer-related death in women worldwide (1). Based on the hormone receptor (estrogen receptor [ER] or progesterone receptor [PR]) and human epidermal growth factor receptor 2 (HER2) status, breast cancer is classified into four main subtypes; hormone receptor-positive/HER2-negative (or luminal A), hormone receptor-positive/HER2-positive (or luminal B), hormone receptor-negative/HER2-positive (HER2-positive or HER2-enriched), and triple-negative breast cancer (TNBC), and the choice of systemic treatment depend on the cancer subtypes (2, 3). In recent decades, there has been remarkable progress in the development of novel molecular targeted therapies (i.e., cyclin-dependent kinase 4 and 6 inhibitors, poly (ADP-ribose) polymerase inhibitors, and HER2 or trophoblast cell surface antigen-2 antibody-drug conjugates) and immunotherapies (i.e., programmed cell death-1 [PD-1] inhibitors) for patients with breast cancer who failed primary systemic treatment with endocrine therapy or chemotherapy (4). Despite significant improvements in therapies, a considerable number of patients still experience recurrence or metastasis after initial therapy, which is responsible for the high mortality in breast cancer (5–7).

Tumor progression and metastasis are driven by the complex interplay between malignant tumor cells and the surrounding non-malignant stroma, comprising the extracellular matrix (ECM), stromal cells such as endothelial cells (ECs), fibroblasts, and infiltrating immune cells (8, 9). Matrix metalloproteinases (MMPs) are zinc-dependent endopeptidases that can degrade and remodel the ECM (10). Studies have identified 23 MMPs in humans (11), and they are generally categorized as collagenases (MMP-1 [interstitial collagenase], -8 [neutrophil collagenase], -13 [collagenase-3], and -18 [collagenase-4]), gelatinases (MMP-2 [72 kDa gelatinase/type IV collagenase], and -9 [92 kDa gelatinase/type IV collagenase]), stromelysins (MMP-3 [stromelysin-1], -10 [stromelysin-2], and -11 [stromelysin-3]), matrilysins (MMP-7 [matrilysin], and -26 [endometase or matrilysin-2]), membrane-type (MMP-14 [MT1-MMP], -15 [MT2-MMP], -16 [MT3-MMP], -17 [MT4-MMP], -24 [MT5-MMP], and -25 [MT6-MMP]), or other miscellaneous types based on substrate specificity or domain structure (12, 13). MMPs, which are produced by non-malignant stromal cells as well as tumor cells, contribute to tumor progression by regulating tumor growth, invasion, angiogenesis, and metastasis (11, 14–16). Although MMPs were initially thought to promote tumor progression by remodeling the ECM through their proteolytic activity, accumulating evidence has provided a novel understanding of the roles and mechanisms of MMPs in cancer. Some MMPs, such as MMP-3 and -8, exhibit protective roles in cancer, and the same MMP can be pro-tumorigenic or anti-tumorigenic depending on the cell type in which it is expressed or the stage of cancer (12, 17, 18). Moreover, MMPs produced from non-malignant cells within the tumor microenvironment (TME) play crucial roles in cancer progression and metastasis. Stromal MMPs, such as MMP-9 and -12, particularly those produced by infiltrating inflammatory cells, have been shown to play anti-tumorigenic roles (17), highlighting the need for further studies on cell type-specific roles of MMPs. MMPs also contribute to tumor progression through their non-catalytic functions (14, 15). Accumulating evidence supports the involvement of MMPs in additional biological processes, such as cell signaling and immune regulation, independent of proteolytic activity (11, 15). In particular, some MMPs, such as MMP-2 and -9, have been demonstrated to influence cancer progression by modulating immune responses in the TME (19, 20). In addition, their biological roles are mediated by non-ECM substrates such as cell surface receptors, growth factors, cytokines, chemokines, and nuclear proteins, as well as ECM substrates. For example, MMP-1 derived from fibroblasts was found to promote the growth and invasion of breast cancer cells by activating protease-activated receptor 1 (PAR1) (21). Macrophage-derived MMP-12 inactivates Glu-Leu-Arg (ELR)1 CXC chemokines, which are involved in neutrophil recruitment to sites of injury or inflammation, and generates C-C chemokine receptor (CCR) antagonists by inactivating monocyte chemotactic proteins, indicating the role of MMP in the regulation of inflammatory response by proteolysis of chemokines (22). While most MMPs are secreted proteases and are recognized to have extracellular activity, recent findings revealed that some MMPs, including MMP-1, -2, -3, -7, -8, -9, -10, -11, -12, -14, -23, and -26, have intracellular functions, suggesting that intracellular and extracellular MMPs are likely to play roles in cancer progression (23).

A subset of MMPs is frequently overexpressed in breast cancer and is associated with prognosis; thus, they have been considered promising therapeutic targets for breast cancer (10, 24, 25). However, while there is a MMP inhibitor approved by the U.S. Food and Drug Administration for treating periodontitis, no MMP inhibitor has been approved for the treatment of cancer (26–28). This may be partly attributable to the lack of understanding of the cell type-specific roles of individual MMP in breast cancer. In this review, the current understanding of the roles of tumoral and stromal MMPs in breast cancer progression is summarized, and recent efforts or advances in the development of highly selective MMP inhibitors are discussed.

## Prognostic values of MMPs in breast cancer

In breast cancer, several MMPs are expressed in various cell types comprising the TME, such as fibroblasts, immune cells, and tumor cells, and their expression at the gene or protein level has been reported to be significantly associated with the prognosis of breast cancer patients (**Table 1**).

**Table 1:** Prognostic significance of MMPs in breast cancer.

MMP	Gene or protein level	Samples (method)	Association of stromal or tumoral MMP expression with clinical outcomes	References
MMP-1 (Interstitial collagenase)	Gene expression	Tissue (Public database analysis)	High → poor prognosis (shorter OS)	(29)
	Gene expression	Tissue (Public database analysis)	High → poor prognosis (shorter brain metastasis-free survival)	(30)
	Gene expression	Tissue (Public database analysis)	High → poor prognosis (shorter recurrence-free survival)	(31)
	Protein	Tissue (IHC)	Positive (fibroblasts) → poor prognosis (shorter relapse-free survival)	(32)
	Protein	Tissue (IHC)	High (tumor cells) → poor prognosis (shorter breast cancer-specific survival)	(33)
	Protein	Tissue (IHC)	High → poor prognosis (shorter disease-free survival) in TNBC	(34)
	Protein	Serum (ELISA)	Low → poor prognosis	(35)
MMP-2 (72 kDa gelatinase/type IV collagenase)	Gene expression	Tissue (Public database analysis)	High → favorable prognosis (longer recurrence-free survival)	(31)
	Protein	Tissue (IHC)	Positive (tumor cells) → poor prognosis (shorter recurrence-free survival or OS)	(36)
	Protein	Tissue (IHC)	High (tumor cells) → poor prognosis (shorter relapse-free survival)	(37)
	Protein	Tissue (IHC)	No significant association of tumoral or stromal MMP2 expression with relapse-free survival	(32)
	Protein	Tissue (IHC)	No significant association of tumor-derived MMP2 expression with OS	(38)
	Protein	Tissue (IHC)	High (tumor cells) → high risk of bone metastasis of breast cancer	(39)
	Protein	Tissue (IHC)	No significant association of tumoral MMP2 with OS MMP2 (stromal cells) → poor prognosis (shorter OS)	(40)
	Protein (Systemic review and meta-analysis)	Tissue (IHC)	High (tumor cells) → poor prognosis (shorter OS)	(41)
	Protein	Serum (ELISA)	High → poor prognosis (shorter disease-free survival and OS)	(42)
	Protein	Serum (ELISA)	High → poor prognosis (shorter disease-free survival)	(43)
MMP-7 (Matrilysin)	Gene expression	Tissue (Public database analysis)	High → poor prognosis (shorter recurrence-free survival)	(31)
	Protein	Tissue (IHC)	Positive (fibroblasts) → poor prognosis (shorter relapse-free survival) Positive (MICs) → poor prognosis (shorter relapse-free survival)	(32)
MMP-8 (Neutrophil collagenase)	Gene expression	Tissue (Public database analysis)	High → favorable prognosis (longer recurrence-free survival)	(31)
	Gene expression	Tissue (qRT-PCR)	Positive (tumor cells) → favorable prognosis (longer relapse-free survival)	(44)
MMP-9 (92 KDa gelatinase/type IV collagenase)	Gene expression	Tissue (Public database analysis)	High → poor prognosis (shorter OS)	(29)
	Gene expression	Tissue (Database analysis)	High → poor prognosis (shorter disease-specific survival)	(45)
	Gene expression	Tissue (Public database analysis)	High → poor prognosis (shorter recurrence-free survival)	(31)
	Gene expression	Tissue (Public database analysis)	High → poor prognosis (shorter brain metastasis-free survival)	(46)
	Protein	Tissue (IHC)	High (tumor cells) → favorable prognosis (longer recurrence-free survival) Positive (stromal cells) → poor prognosis (shorter recurrence-free survival and breast cancer-related survival) in ER-positive breast cancer	(47)
	Protein	Tissue (IHC)	High (tumor cells) → poor prognosis (shorter relapse-free survival)	(37)
	Protein	Tissue (IHC)	Positive (stromal cells) → poor prognosis (shorter relapse-free survival and OS)	(48)
	Protein	Tissue (IHC)	Positive (tumor cells) → poor prognosis (shorter relapse-free survival) Positive (fibroblasts) → poor prognosis (shorter relapse -free survival) Positive (MICs) → poor prognosis (shorter relapse-free survival)	(32)
	Protein	Tissue (IHC)	Positive (fibroblasts) → poor prognosis (shorter relapse-free survival) in luminal A breast cancer Positive (MICs) → poor prognosis (shorter relapse-free survival) in HER2-positive breast cancer	(49)
	Protein	Tissue (IHC)	Positive (tumor cells) → poor prognosis (shorter breast cancer-specific survival)	(50)
	Protein (Systemic review and meta-analysis)	Tissue (IHC)	High (tumor cells) → poor prognosis (shorter OS)	(41)
	Serum	Serum (ELISA)	High → poor prognosis (shorter relapse-free survival and OS)	(51)
MMP-11 (Stromelysin-3)	Gene expression	Tissue (Public database analysis)	High → poor prognosis (shorter disease-specific survival)	(45)
	Gene expression	Tissue (Public database analysis)	High → poor prognosis (shorter disease-free survival and disease-specific survival)	(52)
	Gene expression	Tissue (qRT-PCR)	High → poor prognosis (shorter distant metastasis-free survival)	(53)
	Protein	Tissue (IHC)	Positive (fibroblasts) → poor prognosis (shorter relapse-free survival) Positive (MICs) → poor prognosis (shorter relapse-free survival)	(32)
	Protein	Tissue (IHC)	Positive (tumor cells) → poor prognosis (shorter OS, but not disease-free survival) Positive (stromal fibroblast-like cells) → poor prognosis (shorter disease-free survival and OS)	(54)
	Protein	Tissue (IHC)	High (endothelial cells) → poor prognosis (shorter relapse-free survival and OS)	(55)
	Protein	Tissue (IHC)	Positive (MICs) → poor prognosis (shorter relapse-free survival) in all subtypes of breast cancer Positive (fibroblasts) → poor prognosis (shorter relapse-free survival) in luminal A breast cancer	(49)
	Protein	Tissue (IHC)	No significant association of tumoral MMP-11 expression with prognosis Positive (fibroblasts) → poor prognosis (shorter relapse-free survival and OS) Positive (MICs) → poor prognosis (shorter relapse-free survival and OS)	(56)
	Protein	Tissue (IHC)	High (tumor cells) → poor prognosis (shorter disease-free survival and disease-specific survival)	(52)
	Protein	Tissue (IHC)	No significant association of tumoral MMP-11 expression with prognosis No significant association of MMP-11 expression in fibroblasts with prognosis Positive (MICs) → poor prognosis (shorter distant metastasis-free survival, disease-free survival, and OS)	(57)
MMP-12 (Macrophage metalloelastase)	Gene expression	Tissue (Public database analysis)	High → poor prognosis (shorter OS)	(29)
	Gene expression	Tissue (Public database analysis)	High → poor prognosis (shorter recurrence-free survival and OS)	(31)
MMP-13 (Collagenase-3)	Protein	Tissue (IHC)	Positive (fibroblasts) → poor prognosis (shorter relapse-free survival) Positive (MICs) → poor prognosis (shorter relapse-free survival)	(32)
	Protein	Tissue (IHC)	High (tumor cells) → shorter OS No significant association of stromal fibroblast-derived MMP-13 expression with prognosis	(38)
MMP-14 (MT1-MMP)	Gene expression	Tissue (Public database analysis)	High → poor prognosis (shorter OS)	(29)
	Gene expression	Tissue (qRT-PCR)	High → poor prognosis (shorter metastasis-free survival)	(58)
	Protein	Tissue (IHC)	Positive (MICs) → poor prognosis (shorter relapse-free survival)	(32)
	Protein	Tissue (IHC)	No significant association of tumoral MMP-14 expression with prognosis No significant association of MMP-14 expression in fibroblasts with prognosis Positive (MICs) → poor prognosis (shorter relapse-free survival)	(59)
	Protein	Tissue (IHC)	Positive (MICs) → poor prognosis (shorter relapse-free survival) in luminal B breast cancer	(49)
MMP-15 (MT2-MMP)	Gene expression	Tissue (Public database analysis)	High → poor prognosis (shorter OS)	(29)
	Gene expression	Tissue (Database analysis)	High → poor prognosis (shorter disease-specific survival)	(45)
	Gene expression	Tissue (Public database analysis)	High → poor prognosis (shorter recurrence-free survival and OS)	(31)
MMP-26 (Endometase or matrilysin-2)	Gene expression	Tissue (Public database analysis)	High → favorable prognosis (longer recurrence-free survival)	(31)
	Protein	Tissue (IHC)	High → favorable prognosis (longer disease-free survival and OS)	(60)

ELISA, enzyme-linked immunosorbent assay; ER, estrogen receptor; HER2, human epidermal growth factor receptor 2; IHC, immunohistochemistry; MICs, mononuclear inflammatory cells; OS, overall survival; qRT-PCR, quantitative real-time reverse transcription-PCR.

### Prognostic significance of MMP gene expression

Analyses of publicly available gene expression databases or datasets revealed that high expression of MMP-1, -7, -9, -11, -12, -14, and -15 was significantly associated with decreased patient survival (29, 31, 45). In contrast, high MMP-2, -8, and -26 gene expressions were found to be correlated with a favorable prognosis (31). Quantitative real-time reverse transcription-PCR (qRT-PCR) analyses further confirmed that upregulated MMP-14 transcript levels correlate with shorter metastasis-free survival in breast cancer (58) and that high MMP-11 mRNA levels independently predict the increased risk of distant metastasis in breast cancer, especially in HER2-positive breast cancer (53). Moreover, based on the prognostic significance of their gene expression, some MMPs are included in the prognostic gene signatures of commercial multigene assays for predicting the risk of recurrence in early breast cancer. Seventy gene signatures used to develop MammaPrint (Agendia Inc., Irvine, CA, USA) contain MMP9 as a prognostic gene (61). MMP11 is included in the 21-prognostic gene panel of the Oncotype DX assay (Genomic Health Inc., Redwood City, CA, USA) (62) and 50 genes were used to calculate the risk or recurrence score of the Prosigna PAM50 assay (Veracyte, South San Francisco, CA, USA; formerly: NanoString Technologies, Seattle, WA, USA) (63, 64).

### Prognostic significance of tumoral or stromal MMP protein expression

Numerous immunochemical studies have validated the prognostic significance of the tumoral or stromal MMP expression in breast cancer. Several studies have shown a significant association between stromal MMP-11 or -14 expression and poor prognosis in breast cancer. In particular, MMP-11 expression in mononuclear inflammatory cells (MICs) has been consistently demonstrated to be a strong predictor of decreased survival in patients with breast cancer (49, 56, 57), whereas there have been conflicting reports on the prognostic value of MMP-11 expression in cancer cells or cancer-associated fibroblasts (CAFs) (52, 54, 56, 57). MMP-11 was also found to be expressed in ECs from breast cancer samples, and MMP-11 expression in ECs was significantly correlated with shorter relapse-free and overall survival (OS) (55). MMP-14 expression in MICs is also a negative prognostic factor for relapse-free survival in breast cancer (32, 49, 59).

In cases of MMP-1, -2, -9, and -13, conflicting results on the prognostic values of their tumoral or stromal expression have been reported. While one study showed the prognostic significance of fibroblastic MMP-1 expression (32), another study reported that stromal MMP-1 expression is not significant and that high MMP-1 expression in tumor cells is an independent negative prognostic factor for breast cancer-specific survival (33). Some studies have shown that tumoral MMP-2 expression is significantly associated with shorter survival in patients with breast cancer (36, 37). In particular, high tumoral MMP-2 expression was significantly correlated with an increased risk of bone metastasis in breast cancer (39). A recent systematic review and meta-analysis also confirmed that tumoral MMP-2 overexpression is associated with shorter OS and a higher risk of distant metastasis, suggesting that tumoral MMP-2 expression is a promising negative prognostic factor (41). However, several studies have reported no relationship between MMP-2 protein expression and patient survival (32, 38). In addition, Min et al. reported that stromal MMP-2 expression is an independent factor indicating poor prognosis, whereas tumoral MMP-2 alone has no prognostic value (40). As for MMP-9, several studies have demonstrated that its positive or high expression in tumor cells or stromal cells is a negative prognostic factor for breast cancer (32, 37, 48, 50, 65). A recent study revealed that high MMP-9 expression in tumor cells is significantly associated with poor breast cancer-specific survival and is an independent negative prognostic factor, whereas stromal MMP-9 expression exhibited similar trends but is marginally significant, highlighting that tumoral MMP-9 expression is a more significant predictor of patient survival (50). A recent systematic review and meta-analysis also demonstrated that tumoral MMP-9 overexpression correlates with lymph node metastasis and predicts shorter OS in breast cancer patients (41). In contrast, one study reported that low protein expression of MMP-9 in cancer cells is an independent predictor of shorter recurrence-free survival in patients with breast cancer, indicating that high tumoral MMP-9 expression is a favorable prognostic factor, while stromal MMP-9 expression predicts shorter recurrence-free survival in ER-positive breast cancer (47). Conflicting results regarding the prognostic significance of MMP-13 have also been reported. One study showed a significant association between stromal MMP-13 expression and poor prognosis (32) but another study showed that tumoral MMP-13, not stromal fibroblast-derived MMP-13, correlated with aggressive tumor phenotypes and was an independent negative prognostic factor for OS in breast cancer (38). Further validation of the prognostic value of stromal or tumoral MMP-1, -2, -9, and -13 expression in breast cancer is required.

Consistent with the gene expression data analysis, positive MMP-8 (44) and high MMP-26 protein expression (60) in breast tumor tissues correlated with longer patient survival, indicating that they are favorable prognostic factors in breast cancer.

### Serum MMP levels as prognostic factors

It is notable that serum levels of some MMPs, such as MMP-1, -2, and -9, are associated with prognosis in breast cancer. Serum MMP-9 levels were significantly upregulated in breast cancer patients compared to normal controls and high serum MMP-9 levels were significantly associated with poor prognostic factors, such as higher tumor size and lymph node metastasis, and lower relapse-free survival and OS rates (51). Postoperative high serum MMP-2 levels remain an independent predictor of poor prognosis in node-positive breast cancer, whereas serum MMP-9 levels do not correlate with patient survival (42). Similarly, another study showed that high preoperative serum levels of MMP-2 were associated with shorter disease-free survival in patients with breast cancer in ER-negative, higher histologic grade, or higher nuclear grade breast cancers (43). In contrast, serum MMP-1 levels were significantly lower in patients with breast cancer than in healthy controls, and low serum MMP-1 levels were associated with shorter survival (35). These results suggest that circulating MMP can also be used as a prognostic factor for breast cancer. However, further validation of the clinical utility of circulating MMP levels is warranted.

## The roles of tumoral or stromal MMPs in breast cancer

**Figure 1:**
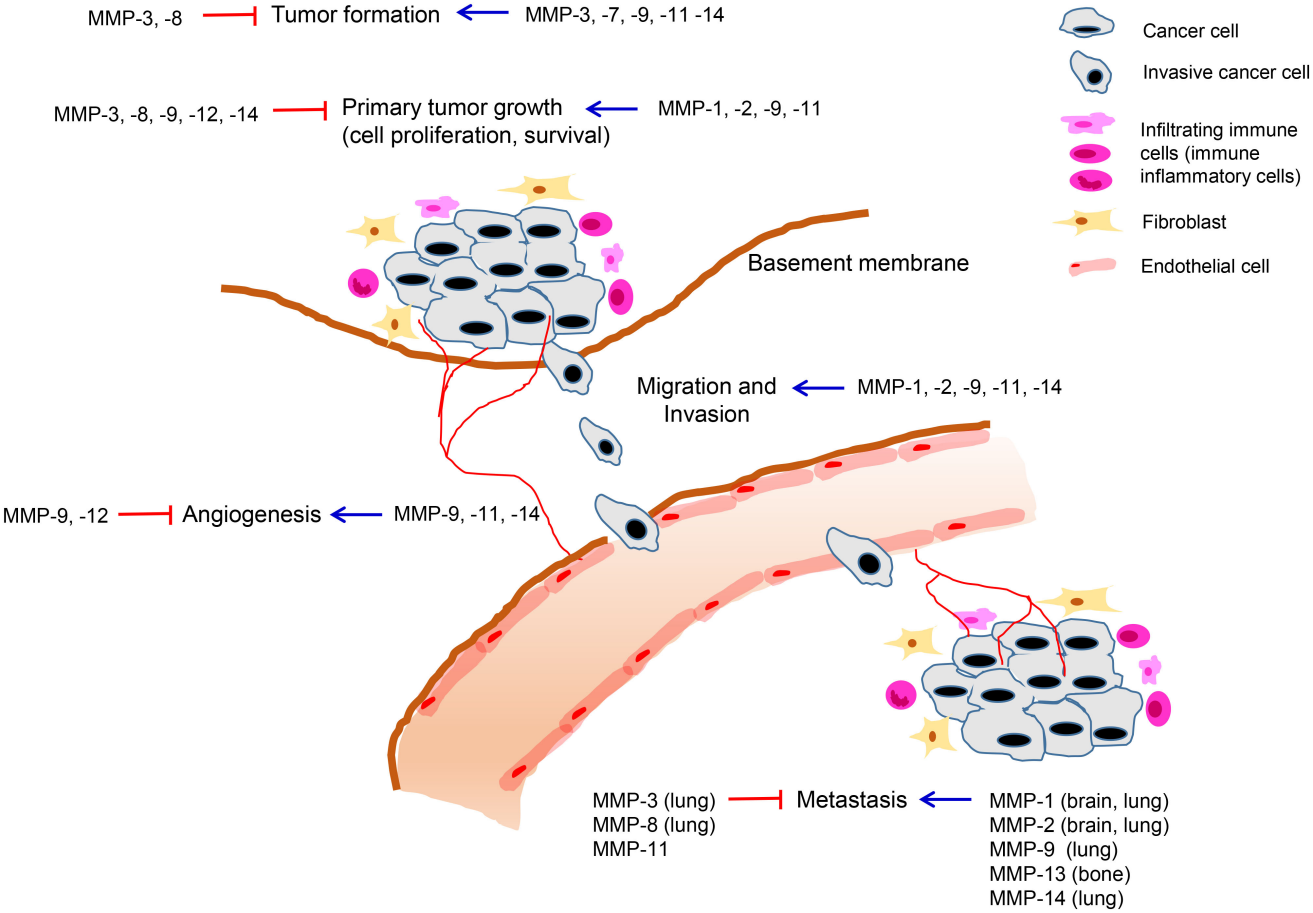
Roles of MMPs in breast cancer development and progression. MMPs are expressed in various cell types including tumor cells and neighboring stromal cells, such as fibroblasts, immune cells, and endothelial cells comprising the tumor microenvironment. Tumoral or stromal MMPs play roles in multiple stages of breast cancer progression including primary tumor growth, angiogenesis, invasion, and metastasis. MMPs can exert pro-tumorigenic or anti-tumorigenic functions in breast cancer and the same MMP can exhibit opposing roles depending on stage of cancer progression.

The role of MMPs in breast cancer progression has been investigated based on their overexpression and prognostic significance. Some MMPs have been shown to promote tumor growth through the regulation of cell proliferation, apoptosis, angiogenesis, or metastasis, while some MMPs have been found to exert both pro-tumorigenic and anti-tumorigenic roles depending on the cell types in which they are expressed or the stages of the disease (**Table 2**, **Figure 1**). Furthermore, a few MMPs have exhibited anti-tumorigenic functions in breast cancer (**Table 2**, **Figure 1**).

**Table 2:** The roles of tumoral or stromal MMPs in breast cancer.

MMP	Roles	Functions	References
MMP-1 (Interstitial collagenase)	Tumoral MMP-1 → Pro-tumorigenic	Promote tumor growth and brain/lung metastasis (*in vivo*)	(66)
	Tumoral MMP-1 → Pro-tumorigenic	Promote brain metastasis (*in vivo*)	(30)
	Tumoral MMP-1→ Pro-tumorigenic	Promote the proliferation, migration and invasion of breast cancer cells (*in vitro*)	(67)
	Tumoral MMP-1→ Pro-tumorigenic	Promote the migration and invasion of breast cancer cells (*in vitro*) Promote lung metastasis (*in vivo*)	(34)
MMP-2 (72 kDa gelatinase/type IV collagenase)	Tumoral MMP-2 → Pro-tumorigenic	Promote the invasion of breast cancer cells (*in vitro*) Promote primary tumor growth and metastasis (*in vivo*)	(68)
	Tumoral MMP-2 → Pro-tumorigenic	Promote brain metastasis (*in vivo*)	(69)
	Tumoral MMP-2 → Pro-tumorigenic	Promote the migration and invasion of breast cancer cells (*in vitro*)	(39)
	Tumoral MMP-2 → Pro-tumorigenic	Promote the migration and invasion of breast cancer cells (*in vitro*)	(70)
	Stromal (fibroblasts) MMP-2 → Pro-tumorigenic	Promote lung metastasis outgrowth (*in vivo*)	(71)
MMP-3 (Stromelysin-1)	MMP-3 →Pro-tumorigenic	Promote tumor formation (*in vivo*)	(72)
	MMP-3 → Pro-tumorigenic	Promote tumor formation (*in vivo*)	(73)
	MMP-3 → Anti-tumorigenic	Suppress tumor formation (*in vivo*)	(74)
	Stromal MMP3 → Anti-tumorigenic	Suppress primary and lung metastatic tumor growth (*in vivo*)	(75)
MMP-7 (Matrilysin)	Tumoral MMP-7 → Pro-tumorigenic	Enhance mammary gland proliferation and tumorigenesis (tumor development) (*in vivo*)	(76)
	Tumoral MMP-7 → Pro-tumorigenic	Promote tumor formation (*in vivo*)	(77)
MMP-8 (Neutrophil collagenase)	MMP-8→Anti-tumorigenic	Suppress tumorigenesis (tumor onset and growth) and lung metastasis (*in vivo*)	(78)
MMP-9 (92 kDa type IV collagenase)	Tumoral MMP-9 (secreted MMP-9 from tumor cells) → Pro-tumorigenic	Promote tumor growth and angiogenesis (*in vivo*)	(79)
	Tumoral MMP-9 → Pro-tumorigenic (Pro-metastatic)	Promote the invasion of breast cancer cells (*in vitro*) Promote lung metastasis (*in vivo*)	(80)
	MMP-9 → Pro-tumorigenic	Promote tumor onset in C3 (1)-Tag model but no effect in MMTV-Neu model (*in vivo*)	(81)
	Stromal MMP-9 → Pro-tumorigenic (Pro-metastatic)	Promote lung metastasis in C57BL/6 background mice but no effect in FVB/N background mice (*in vivo*)	(82)
	MMP-9→Anti-tumorigenic	Suppress tumor growth rate and angiogenesis (*in vivo*)	(83)
	MMP-9→Anti-tumorigenic	Suppress tumor growth rate and neoangiogenesis (*in vivo*)	(19)
MMP-11 (Stromelysin-3)	Tumoral MMP-11 →Pro-tumorigenic	Increase the survival of breast cancer cells (*in vitro*)	(84)
	Tumoral MMP-11 →Pro-tumorigenic	Enhance tumorigenesis (tumor onset and growth) (*in vitro and in vivo*)	(85)
	Stromal MMP-11 → Pro-tumorigenic for primary tumor growth but anti-tumorigenic for metastasis (Anti-metastatic)	Promote tumor formation and primary tumor growth but suppress metastasis (*in vivo*)	(86)
	Stromal (macrophages) MMP-11 → Pro-tumorigenic	Promote the migration of breast cancer cells, recruitment of monocyte and endothelial cell tube formation (*in vitro*)	(57)
MMP-12 (Macrophage metalloelastase)	Tumoral MMP-12 → Anti-tumorigenic	Suppress angiogenesis and tumor growth (*in vitro and in vivo*)	(87)
MMP-13 (Collagenase-3)	Tumoral MMP-13 → Pro-tumorigenic	Promote bone destruction in metastatic region (metastatic osteolytic lesions) (*in vivo*)	(88)
	MMP-13 → Pro-tumorigenic	Promote tumor-induced osteolysis (bone destruction) (*in vivo*)	(89)
MMP-14 (MT1-MMP)	Tumoral MMP-14 → Pro-tumorigenic (Pro-metastatic)	Promote the invasion of breast cancer cells (*in vitro*) Promote lung metastasis (*in vivo*)	(90)
	Tumoral MMP-14 → Pro-tumorigenic	Promote invasion (*in vivo*)	(58)
	Tumoral MMP-14 → Pro-tumorigenic	Promote local invasion and metastasis (*in vivo*)	(91)
	Tumoral MMP-14 → Pro-tumorigenic	Promote the characteristics of tumor-initiating cells and tumorigenicity (tumor onset and growth) (*in vitro and in vivo*)	(92)
	Stromal MMP-14 → Anti-tumorigenic for primary tumor growth but pro-tumorigenic for metastasis (Pro-metastatic)	Suppress primary tumor growth but promote lung metastasis (*in vivo*)	(93)
	Stromal MMP-14 → Pro-tumorigenic	Promote the migration of breast cancer cells (*in vitro*)	(94)
	Stromal (endothelial cells) MMP-14 → Pro-tumorigenic	Promote the adhesion of extracellular matrix and endothelial cell tube formation (*in vitro*)	(95)

### Pro-tumorigenic MMPs

Consistent with the significant association of their high expression in tumor cells or stromal cells with poor clinical outcomes, MMP-1, -2, -7, -13, and -14 exhibited pro-tumorigenic functions in breast cancer.

Breast cancer is the second most common cancer that metastasizes to the brain after lung cancer, and among the breast cancer subtypes, TNBC and HER2-positive breast cancer have a higher risk of brain metastasis than other subtypes (96, 97). MMP-1 has been shown to play a role in breast cancer brain metastasis. Knockdown of MMP-1 expression in TNBC cells significantly inhibited breast cancer growth and brain metastasis in a xenograft model, indicating that tumoral MMP-1 promotes tumor growth and formation of brain metastasis in TNBC (66). Similarly, another study analyzing the clinical significance of MMPs in brain metastasis-free survival of breast cancer using a public gene expression database found that high MMP-1 gene expression is positively correlated with brain metastasis of breast cancer, and it also demonstrated that knockdown of MMP-1 expression in TNBC cells significantly blocked brain metastasis of breast cancer *in vivo* (30). This study further revealed that MMP-1 is highly expressed in brain metastatic breast cancer cells and can degrade key components of the blood-brain barrier, suggesting that MMP-1 secreted from brain metastatic breast cancer cells enhances brain metastasis by increasing the permeability of the blood-brain barrier. Decreased MMP-1 expression in luminal A (MCF-7) and TNBC (MDA-MB-231) cells also significantly inhibited cell proliferation, migration, and invasion *in vitro* (67). A recent study revealed that exosomal MMP-1 secreted from TNBC cells increased their migration and invasion and further enhanced their metastatic potential *in vivo* (34). These results provide evidence that MMP-1 secreted from tumor cells has a tumor-promoting role in breast cancer; in particular, it promotes brain metastasis in TNBC. MMP-1 was also reported to be involved in tamoxifen resistance in breast cancer. Downregulation of MMP1 in tamoxifen-resistant breast cancer cells induced tamoxifen sensitivity *in vitro* and retarded tumor growth *in vivo* (98).

The tumor-promoting functions of tumoral and stromal MMP-2 have also been demonstrated. Tumoral MMP-2 enhanced tumor growth and metastasis in an orthotopic mouse model of breast cancer (68, 69). Notably, similar to MMP-1, these studies showed that tumoral MMP-2 promotes breast cancer metastasis to the brain. Moreover, high MMP-2 expression in tumor cells was correlated with an increased risk of bone metastasis in breast cancer patients, and MMP-2 overexpression promoted the migration and invasion of breast cancer cells (39). Another *in vitro* study showed that PAR2-induced MMP-2 expression promotes the migration of human breast cancer cells through the p38 MAPK/MK2/HSP27 axis (70). On the other hand, host-derived MMP-2, which is mainly produced from stromal fibroblasts, was also shown to promote the outgrowth of mammary tumors in the lungs *in vivo* (71). These results suggest that both tumoral and stromal MMP-2 are involved in the promotion of breast cancer progression.

MMPs can activate other MMPs by converting their inactive form to active form (12). MMP-7 activates gelatinases, including MMP-2 and -9, thereby enhancing their proteolytic activity (99). MMTV-Neu transgenic mice that express MMP-7 in the mammary epithelium exhibited enhanced mammary gland proliferation and tumorigenicity (76). Another study showed that MMP-7 promotes mammary epithelial cell tumorigenesis through the receptor tyrosine-protein kinase ErbB-4 (77), further supporting the hypothesis that MMP-7 contributes to early-stage mammary tumorigenesis.

MMP-13 is associated with breast cancer-induced osteolysis, suggesting that MMP-13 is a promising therapeutic target for breast cancer bone metastasis (88, 89). MMP-13 expression was found to be upregulated in bones of a metastatic TNBC mouse model (88) or at the tumor-bone interface from syngeneic mice injected with mammary tumor cell lines with metastatic potential (89). Furthermore, the knockdown of MMP-13 at the tumor-bone interface by treatment with MMP-13 antisense oligonucleotide significantly reduced bone destruction, indicating that MMP-13 contributes to breast cancer-induced osteolysis (89). In addition, co-culture of breast cancer cells and osteoblasts has revealed that MMP-13 expression can be induced in osteoblasts by soluble factors produced by breast cancer cell lines, suggesting the involvement of osteoblastic MMP-13 in bone metastasis of breast cancer (100).

Upregulation of MMP-14, a membrane-anchored MMP, is associated with an increased risk of metastasis in breast cancer (58, 90, 91). Downregulation of cancer cell-expressed MMP-14 did not affect primary growth but inhibited lung metastasis in an orthotopic mouse model of TNBC (90). In an intraductal xenograft model, MMP-14 was found to be required for cancer progression from carcinoma *in situ* to the invasive stage in basal-like breast cancer (58). Feinberg et al. demonstrated that tumor cell-derived MMP-14, but not stromal MMP-14, promotes local invasion and metastasis *in vivo*, supporting the pro-metastatic role of tumoral MMP-14 in breast cancer (91). The effect of MMP-14 on tumor initiation has also been reported. Reduced MMP-14 expression in tumor-initiating cells from a breast cancer mouse model decreased the characteristics of tumor-initiating cells, delayed tumor onset, and decreased tumor volume, indicating that tumoral MMP-14 promotes tumorigenicity *in vitro* and *in vivo* (92). In contrast, other studies have reported that stromal MMP-14 plays a role in breast cancer. MMP-14 was expressed mainly in the stroma of PyMT-induced tumors, and MMP-14-deficient MMTV-PyMT tumors displayed remarkably reduced lung metastasis compared to wild-type tumors, indicating that stromal MMP-14 is required for lung metastasis in breast cancer (93). Notably, this study revealed faster tumor growth in MMP-14-deficient tumors than in wild-type tumors, indicating an inhibitory role of MMP-14 in primary tumor growth. These results suggested the opposing role of stromal MMP-14 depending on the stage of breast cancer progression. Soluble MMP-14 derived from bone marrow-derived stromal cells promoted the migration of luminal A breast cancer cells by inducing endoglin (transforming growth factor-β [TGF-β] auxiliary receptor) shedding on the breast cancer cell surface, suggesting a pro-tumorigenic role of stromal MMP-14 (94). In particular, MMP-14 plays a crucial role in vessel maturation and angiogenesis associated with cancer progression (101, 102). Selective inhibition of MMP-14 using an anti-MMP-14 antibody impaired the migration, invasion, and tube formation of ECs *in vitro*, in part by blocking MMP-2 activation in ECs (103). MMP-14 in ECs regulated angiogenesis-related functions through the modulation of MMP-2 expression and activity, indicating a role of MMP-14 produced by ECs in angiogenesis (95). Similarly, a recent study demonstrated that loss of EC-derived MMP-14 inhibits melanoma growth and metastasis by regulating tumor vessel stability (104). These results show that both stromal and tumoral MMP-14 may contribute to tumor progression in breast cancer. However, the roles and functions of stromal MMP-14 in breast cancer remain unclear, and further studies are required to elucidate them.

### MMPs with dual or opposing roles

Some MMPs, such as MMP-3, -9, and-11, have exhibited dual roles or conflicting data on their roles in breast cancer progression. Conflicting data regarding the role of MMP-3 in breast cancer have been reported. In some studies, MMP-3 has been reported to stimulate spontaneous tumor formation in the mammary glands of transgenic mice (72, 73), suggesting its tumor-promoting role in breast cancer. In contrast, another study showed that tumor formation was decreased in MMP-3-expressing transgenic mice (74). Moreover, in another study, loss of stromal MMP-3 was found to increase the tumor burden, suggesting that stromal MMP-3 plays a protective role during breast cancer development by inhibiting tumor growth (75).

MMP-9 has also been shown to exert both pro- and anti-tumorigenic roles in breast cancer. Some studies have demonstrated the pro-tumorigenic or pro-metastatic activity of MMP-9 *in vivo*, but its effect varied with different mouse models of breast cancer. An early study using xenograft models of luminal A MCF-7 breast cancer cells showed that secreted MMP-9 promotes tumor growth and angiogenesis (79). In another study using genetically engineered breast cancer mouse models, MMP-9 knockout delayed tumor onset in the basal-like TNBC (C3(1)-Tag) model but had no effect on tumorigenesis in the luminal MMTV-Neu model (81). This difference was related to the protein expression levels of insulin-like growth factor-binding protein-1 (IGFBP-1), which is a MMP-9 substrate. While MMTV-Neu tumors had low levels of IGFBP-1 independent of MMP-9 status, IGFBP1-1 expression was increased in the MMP-9 knockout C3(1)-Tag model compared to the MMP-9 wild-type C3(1)-Tag model, suggesting that the tumor-promoting effect of MMP-9 is dependent on the status of its substrate, IGFBP-1. Tumor cell-produced MMP-9 was shown to promote metastasis in an orthotopic mouse model of basal-like TNBC (80). On the other hand, stromal MMP-9 was also shown to play a role in breast cancer, but the effect of stromal MMP-9 was dependent on the genetic background of the mouse stain. Inhibition of MMP-9 produced predominantly by inflammatory cells in the MMTV-PyVT mouse mammary tumor model reduced lung metastasis without affecting primary tumor growth; however, this effect was only observed in mice with a genetic background derived from C57BL/6, suggesting that the pro-metastatic role of host-derived stromal MMP-9 is dependent on genetic background (82). Taken together, these results illustrate that tumor cell-produced or stromal cell-derived MMP-9 can exert pro-tumorigenic or pro-metastatic roles in breast cancer under certain circumstances.

Studies have also demonstrated the anti-tumorigenic activity of MMP-9 in breast cancer. Adenoviral gene transfer of MMP-9 in MCF-7 tumors in nude mice significantly reduced tumor growth and microvessel area, with increased levels of anti-angiogenic endostatin, indicating that MMP-9 can inhibit angiogenesis by the generation of anti-angiogenic factors (83). Another study also reported decreased tumor growth and angiogenesis by adenoviral gene transfer of MMP-9 in mouse models of human breast cancer (luminal MCF-7 tumors in nude mice and MMTV-PyMT tumors) (19). In particular, this study showed that MMP-9 promotes an anti-tumor immune response by inducing neutrophil infiltration and activating tumor-infiltrating macrophages, suggesting that the anti-tumor activity of MMP-9 is mediated by its modulation of the innate immune response.

MMP-11 has a dual role in breast cancer, depending on the stage of cancer. In a study using a MMTV-Ras transgenic mouse model of breast cancer, tumor formation was delayed and the number and tumor size of the primary tumor were lower, but a higher number of metastases were observed in MMP-11-deficient mice than in wild-type mice, indicating that MMP-11 promotes tumorigenesis in the early stages of breast cancer but inhibits the metastasis of tumor cells in the late stage of breast cancer (86). This study revealed that MMP-11 in the TME has different functions in breast cancer progression. Other studies have shown the tumor-promoting effects of tumoral MMP-11 in breast cancer. MMP-11 overexpression increased the survival of MCF-7 cells (84) and enhanced tumorigenicity *in vitro* (MCF-7 and MDA-MB-231 cells) and *in vivo* (85). However, despite the obvious association of MMP-11 expression in MICs with poor clinical outcomes in patients with breast cancer, the role and molecular mechanisms of stromal MMP-11 in breast cancer remain unclear. Our recent study revealed for the first time that MMP-11 produced by macrophages enhanced the migration of HER2-positive breast cancer cells and recruitment of monocytes through C–C motif chemokine ligand 2 (CCL2)−CCR2 signaling, whereas MMP-11 overexpression in tumor cells did not promote the proliferation or migration of breast cancer cells (57). These results showed that stromal MMP-11 may play a tumor-promoting role in HER2-positive breast cancer by interacting with breast cancer cells and other stromal cells. Importantly, some studies have reported a correlation between MMP gene expression and infiltration of various immune cells in breast cancer, suggesting the involvement of MMPs in the regulation of the TME or immune response (31, 52). In particular, Kim et al. (52) showed that high MMP-11 gene expression is significantly associated with low levels of immune cells, including CD8^+^ T cells, CD4^+^ T cells, and B cells, and is related to low immune response. Furthermore, this study demonstrated the correlation of high MMP-11 expression with low infiltrating CD8^+^ or CD4^+^ T cells using immunohistochemical analysis, suggesting that the reduced anti-tumor immune response by MMP-11 contributes to the promotion of breast cancer progression. However, the mechanistic roles of MMP-11 in the regulation of the immune response in the TME are unclear, and further studies are required to elucidate the immune response-related role of MMP-11 in breast cancer progression.

### Anti-tumorigenic MMPs

It is now evident that some MMPs, such as MMP-8, -12, -19, and -26 have protective roles in cancer progression (12, 105). In breast cancer, MMP-8 and -12 have exhibited anti-tumorigenic effects *in vivo*. Loss of MMP-8 in the MMTV-PyMT transgenic mouse model of human luminal breast cancer promoted tumor onset, growth, and lung metastasis (78), illustrating the suppressive roles of MMP-8 in tumor progression and metastasis in breast cancer. Tumor-derived MMP-12 inhibited angiogenesis *in vitro* and *in vivo* in breast cancer (87).

## The roles of tissue inhibitors of MMPs in breast cancer

The proteolytic function of MMPs is regulated by tissue inhibitors of MMPs (TIMPs) (106). The four mammalian TIMPs (TIMP-1, -2, -3, and -4) are endogenous secreted proteins to inhibit MMPs (107). Each of the TIMPs inhibits specific MMPs, and some MMPs such as MMP-2 and -9 interact with several TIMPs (108). Alterations in the expression of TIMPs have been identified in human cancers and their expression correlates with clinical outcome (107, 108). Given their anti-proteolytic function in ECM, TIMPs were initially thought to play protective roles in cancer progression (108). However, TIMPs have been found to be associated with poor prognosis and exert tumor-promoting functions in some human cancers (107, 108). In addition to interaction with MMPs, TIMPs can bind to other interaction partners such as cell surface receptors, and they have multiple biological functions including cell proliferation, apoptosis, migration, invasion and angiogenesis by MMP-dependent or -independent mechanisms (106, 107, 109).

In breast cancer, TIMP-1 expression in primary tumor tissues is an independent poor prognostic factor (32, 110). Moreover, studies have demonstrated that breast cancer patients with high levels of serum TIMP-1 have a significantly shorter survival (51, 111). Consistent with its prognostic significance, TIMP-1 overexpression was shown to promote MDA-MB-231 tumor growth in SCID mice (112). TIMP-1/CD63 signaling enhanced cell motility through the induction of epithelial−mesenchymal transition phenotypes in human breast epithelial cells (113). TIMP-1 knockdown in TNBC cells induced cell cycle arrest, and Akt signaling pathway was associated with the regulation of cyclin D1 expression by TIMP-1, indicating that TIMP-1 increases TNBC cell proliferation by Akt activation (114). This study further showed that blocking TIMP-1 activity using neutralizing antibody inhibits TNBC cell growth *in vivo*. CAF-derived TIMP-1 was involved in the migration and growth of breast cancer cells though TIMP-1/CD63/ITGB1/STAT3 feedback loop (115). Regarding the prognostic value and role of TIMP-2 and -3 in breast cancer, conflicting results have been reported. High tissue expression levels of TIMP-2 were significantly associated with favorable clinical outcome (116). Several studies have demonstrated tumor suppressive role of TIMP-2 in breast cancer. TIMP-2 adenoviral delivery inhibited tumor growth, angiogenesis and metastasis in MDA-MB-231 breast tumor xenografts (117). In another study, TIMP-2 overexpression delayed growth and angiogenesis of mammary carcinoma *in vivo* in association with downregulation of vascular endothelial growth factor (VEGF) expression, indicating that reduced VEGF expression plays a role in anti-tumorigenic effects of TIMP-2 (118). A recent study also revealed inhibitory effects of TIMP-2 on tumor growth and metastasis in murine model of TNBC (119). Conversely, the correlation of high TIMP-2 levels with poor prognosis in breast cancer has also been reported (32, 120, 121). Tumor cell-derived TIMP-2 was shown to induce endothelial dysfunction and promote transmigration of breast cancer cells across vascular endothelial monolayers through the activation of endothelial MMP-2 in the presence of active MMP-14, suggesting the role of TIMP-2/MMP-14/MMP-2 pathway during metastasis (122). As for TIMP-3, stromal TIMP-3 expression is a poor prognostic factor in breast cancer (32), while high TIMP-3 mRNA levels are associated with favorable prognosis (123). TIMP-3 overexpression inhibited cell growth and induced apoptosis in breast cancer cells *in vitro* (124). In contrast, *Timp3* loss suppressed tumorigenesis in mouse models of human breast cancer, suggesting the tumor-promoting role of TIMP-3 *in vivo* (125–127). TIMP-4 expression correlated with poor clinical outcome in early breast cancer (126). Treatment with recombinant TIMP-4 significantly stimulated the growth of MDA-MB-231 cells (127). Further studies to elucidate the mechanistic roles and functions of TIMPs in breast cancer are warranted.

## Therapeutic targeting of MMPs in breast cancer

Several MMP inhibitors have been developed and tested for various cancers based on their prognostic significance and promising preclinical data (16, 27). Early clinical trials with broad-spectrum MMP inhibitors (such as batimastat and marimastat) were unsuccessful because of poor bioavailability, lack of survival benefits, and toxicity (16, 27). The main reasons for these failures have been attributed, in part, to a poor understanding of the roles of MMPs, lack of drug specificity, and inappropriate clinical trial design (11, 12, 16). As some MMPs have protective roles in cancer progression, the negative results of early clinical trials with broad-spectrum of MMP inhibitors were thought to be related to the effects of broad-spectrum MMP inhibitors on anti-tumorigenic MMPs (12, 16). Given that MMP inhibitors are likely to be effective in the early or pre-metastatic stages based on their preclinical data, the design of previous clinical trials of MMP inhibitors tested in patients with advanced or metastatic cancer may be inappropriate (16). Moreover, MMP inhibitor-related toxicities such as musculoskeletal toxicity are assumed to be related to the poor specificity of MMP inhibitors (11, 16). Therefore, substantial efforts have been made to develop more specific MMP inhibitors. However, clinical trials for small molecule-based specific MMP inhibitors (such as tanomastat, prinomastat, and rebimastat), which still target multiple MMPs, have also been halted or canceled owing to negative results (16). Since then, attempts have been made to develop highly specific MMP inhibitors that target a single MMP using novel approaches. In a phase III trial of the broad-spectrum MMP inhibitor marimastat in metastatic breast cancer, marimastat did not prolong progression-free survival (128). Phase II small pilot trials of adjuvant marimastat and rebimastat in early-stage breast cancer were performed; however, further clinical trials with these MMP inhibitors in adjuvant settings were not feasible due to failure of chronic administration to reach the target ranges of plasma concentrations and toxicity (129, 130).

Several novel approaches have been attempted for the development of highly selective MMP inhibitors. The non-catalytic domains of MMPs have been targeted. MMPs are composed of various domains, including a signal peptide, propeptide, catalytic domain that binds a Zn residue, and hemopexin (PEX) C-terminal domain, while the domain architecture differs depending on the type of MMP (11, 24). As active sites in the catalytic domain of MMPs are highly conserved (11, 12, 24), MMP inhibitors that target the catalytic domains have the potential to repress multiple MMPs rather than specific MMPs. In contrast, the non-catalytic domains of MMPs, such as the PEX domain, vary between MMPs, and targeting these domains is likely to improve the specificity of MMP inhibitors. In addition, MMPs have been shown to play roles in cancer progression and metastasis through their non-catalytic functions, independent of their proteolytic activity (14, 15). Therefore, targeting the non-catalytic domain of MMPs is a promising approach to develop specific MMP inhibitors. For example, given the pro-tumorigenic role of the PEX domain of MMP-14 through homodimerization or interactions with other molecules, a novel small molecule that specifically inhibits the PEX domain of MMP-14 was identified, which was shown to suppress tumor growth in a xenograft mouse model of breast cancer (131).

Metastatic breast cancer is a major cause of death from breast cancer, and targeting MMPs involved in breast cancer metastasis has also been considered. Breast cancer frequently metastasizes to several organs, including bone, lung, brain, and liver; with bone being the most common metastatic site of breast cancer (97). Accordingly, attempts have been made to develop selective MMP inhibitors that are effective against bone metastatic breast cancer. A MMP-13 selective inhibitor (Cmpd-1), which was identified from a panel of small molecule pyrimidinetrione-based inhibitors (132), was shown to inhibit osteolytic damage and reduce primary tumor growth in a human breast cancer xenograft model of TNBC using MDA-MB-231 cells, suggesting that MMP-13 selective inhibitors reduce tumor-induced bone osteolysis (133). Moreover, bisphosphonic-based MMP-2 inhibitors specifically targeting bones also demonstrated inhibitory effects on tumor growth and tumor-associated bone destruction in bone metastatic mouse models of breast cancer (134)

Currently, MMP inhibitory monoclonal antibodies are considered promising MMP-targeting therapies because they have higher target selectivity and better pharmacokinetic profiles than small-molecule agents (12, 135). Inhibitory monoclonal antibodies targeting a single MMP, mainly MMP-9 or -14, have been developed and demonstrated anti-tumor activity in preclinical models of breast cancer. A monoclonal antibody against murine MMP-9 (AB0046) decreased primary growth in an orthotopic model of HER2-driven breast cancer (HC11-NeuT) in immunocompetent mice (136). This study also showed that combined treatment with an anti-MMP-9 antibody and immune checkpoint inhibitor (ICI) targeting programmed cell death-ligand 1 (PD-L1), improved anti-tumor immune response to anti-PD-L1 by increasing the levels of T-helper cell 1 type cytokines and infiltration of effector/memory T cells into tumors. This suggested that MMP-9 promotes anti-tumor T cell response. In contrast, in the MMTV-PyMT luminal B breast cancer model, blocking active MMP-9 with a monoclonal antibody suppressed lung metastasis without affecting primary tumor growth (137). As MMP-14 is a membrane-type MMP, and active MMP-14 is located on the cell surface, antibody-mediated therapy can block the function of MMP-14 in cancer. An anti-MMP-14 inhibitory antibody (DX-2400) identified using a human Fab displaying phage library demonstrated inhibitory effect on primary tumor growth and incidence of metastasis to the lung and liver in a MDA-MB-231 orthotopic model (103). Based on the anti-angiogenic activity of DX-2400, this study also evaluated the combination of DX-2400 and bevacizumab (an antibody against VEGF) and found that it retarded tumor growth to a greater extent than treatment with either agent individually in the MDA-MB-231 xenograft model, suggesting a promising approach of anti-angiogenic agents in combination with MMP-14 inhibitor for synergistic anti-tumor effects in breast cancer. DX-2400 was further validated to reduce primary tumor growth in orthotopic murine breast cancer models and was found to improve the response to radiotherapy in MMP-14-high-expressing 4T1 tumors (138). Importantly, this study revealed that tumor growth suppression by MMP-14 blockade was associated with reduced immunosuppressive TGF-β and the shift of macrophages to anti-tumor M1 phenotype, while improved vascular function and tissue oxygenation by MMP inhibition-induced increase in tumor inducible nitric oxide synthase led to increased radiation response in MMP-14-high-expressing tumors. Given that dysregulation of ECM genes linked to the activation of immunosuppressive TGF-β signaling in CAFs predicts a failure to PD-1 blockade (139), MMP-14 inhibitory antibody inhibiting TGF-β signaling is likely to improve the response to ICI. Another MMP-14-selective antibody (Fab 3A2) was also identified by screening human Fab fragment libraries carrying long convex-shaped paratopes to overcome limitations in physical access of the antibody to the active region of MMP-14, which is concave-shaped. Fab 3A2 was found to inhibit protease activity of the MMP-14 by accessing the convex pocket of MMP-14 (140). Moreover, a recombinant human IgG specific to MMP-14 (IgG 3A2) demonstrated inhibitory effects on the growth of the primary tumor and lung metastasis in the 4T1 highly metastatic, syngeneic, orthotopic model of breast cancer (141). MMP-14-specific Fabs were also identified using phage-displayed synthetic humanized Fab library against the extracellular domain of MMP-14, and among them, Fab 3369, which inhibits the catalytic domain of MMP-14, demonstrated anti-tumor activities *in vitro* and *in vivo* (142). Fab3369 significantly decreased the invasion of TNBC cells *in vitro* and suppressed tumor growth and metastasis in TNBC mouse models (mammary orthotopic tumor xenografts and syngeneic 4T1 mammary tumors). Importantly, its *in vivo* anti-tumor effect was associated with the disruption of immunosuppressive TME, including limiting tumor neoangiogenesis and hypoxia, suggesting that MMP-14 inhibitory antibody suppresses tumor progression and metastasis through its effect on TME in TNBC. Importantly, these promising preclinical data of MMP-9 and -14 inhibitory monoclonal antibodies in HER2-positive breast cancer or TNBC mouse models showed that the anti-tumor effects of MMP inhibitory antibodies are associated with disruption of the immunosuppressive TME, indicating that the use of MMP inhibitors in combination with immunotherapy could improve the efficacy of immunotherapy in breast cancer.

The expression and activity of MMPs are regulated by several mechanisms, including TIMPs (107, 108), noncoding RNAs (143, 144), cell signaling pathways (145–147) or E3 ubiquitin ligases (148) in cancer. MMP inhibitors using TIMPs or E3 ubiquitin ligases are emerging as alternative strategies to develop highly specific MMP inhibitors. A recently developed platform identified soluble TIMP-1 variants that are highly selective for the inhibition of MMP-3 over MMP-10, which is the MMP most similar to MMP-3 in sequence, structure, and function, indicating that this platform and screening strategy can be used to develop selective MMP inhibitors (149). Knockdown of the E3 ligase WSB-1 inhibited the metastatic potential of hormone receptor-negative breast cancer *in vitro* and *in vivo* in association with decreased MMP activity, suggesting that the activity of pro-tumorigenic MMPs can be regulated by E3 ligase (150). In addition, several microRNAs (miRNAs) have been reported to be involved in cancer progression and metastasis through MMP regulation (144). In breast cancer, miR-106b (39) and miR-429 (151) have been shown to play a role in bone metastasis of breast cancer by regulating MMP-2 and -9, respectively. Another study revealed that miR-509 suppressed the invasion and trans-endothelial migration of brain metastatic TNBC cells by inhibiting Rho-mediated MMP-9 expression, and high MMP-9 gene expression is significantly associated with shorter brain-metastasis-free survival in breast cancer patients, indicating that miR-509/Rho/MMP-9 axis is a potential target of brain metastasis of breast cancer (46). These results suggest that targeting miRNAs that regulate MMP expression is a potential avenue to target MMP activity for breast cancer therapy. MMPs have also been shown to be regulated by upstream signaling pathways in breast cancer. For example, upregulation of MMP-14 was blocked by upstream PI3K-AKT dependent β-catenin accumulation, thereby inhibiting the invasion and migration of breast cancer cells (152). Our recent study also showed that CCL2 produced by MMP-11-overexpressing macrophages activates the MAPK pathway through its receptor CCR2 in breast cancer cells, thereby enhancing the migration of HER2-positive breast cancer cells by increasing the expression of MMP-9, illustrating the regulation of MMP-9 by the MAPK pathway (57).

## Conclusions

The failures of early clinical trials evaluating broad-spectrum MMP inhibitors as anti-cancer agents revealed that MMPs can be both drug targets and antitargets depending on the cell type in which they are expressed or the stage of cancer. Despite the disappointing results for early broad-spectrum MMP inhibitors and the anti-tumorigenic roles of some MMPs, selective MMP inhibitors that have been developed recently are still promising strategies for cancer treatment. MMP inhibitory monoclonal antibodies targeting MMP-9 or -14 demonstrated promising anti-tumor activities in preclinical models of breast cancer and may exhibit clinical benefits in cancer patients without significant toxicity in clinical trials. Moreover, their anti-tumor effects were shown to be associated with the modulation of the tumor immune microenvironment, suggesting that a selective MMP inhibitor in combination with immunotherapy could enhance patients’ response to immunotherapy. However, several challenges remain to be resolved. These include: 1) Clinical trials for MMP inhibitors should be appropriately designed considering the stages of cancer in which MMPs are involved. Based on the pro-tumorigenic roles of MMPs in the pre-metastatic stages of cancer, MMP inhibitors are likely to be clinically beneficial in early-stage cancer. 2) Given the broad expression of multiple MMPs in various cell types, including tumor cells, fibroblasts, ECs, and immune cells, local targeted delivery of MMP inhibitors to specific sites may be required. 3) The identification and validation of reliable biomarkers for predicting the efficacy or toxicity of MMP inhibitors are also necessary to accelerate the progress of drug development. 4) Despite the known prognostic significance of tumoral or stromal MMPs in breast cancer, their cell type-specific or stage-specific roles in breast cancer progression and metastasis are not fully understood. Thus, further studies to clarify the mechanistic roles of tumoral or stromal MMPs during cancer progression are required to develop novel MMP inhibitors for breast cancer treatment.

## Author contributions

MK wrote, reviewed and edited the manuscript.
